# Exploring barriers to dementia screening and management services by general practitioners in China: a qualitative study using the COM-B model

**DOI:** 10.1186/s12877-023-03756-x

**Published:** 2023-01-31

**Authors:** Ni Gong, Dan Yang, Jianfeng Zou, Qianyu He, Lei Hu, Weiju Chen, Jing Liao

**Affiliations:** 1 School of Nursing, Jinan University, Guangzhou, Guangdong, 510632 China; 2 Department of Medical Statistics and Epidemiology, School of Public Health, Sun Yat-Sen University, Guangzhou , Guangdong, 510080 China; 3 Longhua District Chronic Disease Control Center, Longhua District, Shenzhen , Guangdong, 518110 China; 4 Sun Yat-Sen Global Health Institute, Institute of State Governance, Sun Yat-Sen University, Guangzhou, Guangdong China

**Keywords:** Dementia screening and management, General practitioner, Capability/Opportunity/Motivation-Behavior model

## Abstract

**Background:**

Dementia has become a global public health problem, and general practitioners (GPs) play a key role in diagnosing and managing dementia. However, in Chinese primary care settings, dementia is underdiagnosed and inefficiently managed, and dementia screening and management services provided by GPs are suboptimal. The reasons underlying this gap are poorly understood. This study aimed to determine the barriers that hinder GPs from actively promoting dementia screening and management, and thereby provide insights for the successful promotion of dementia screening and management services in primary care.

**Methods:**

Purposive sampling was used. And focus groups and in-depth interviews were conducted face-to-face among GPs from community health service centers (CHSCs) in South China. Thematic analysis was used to identify barriers to screening and managing dementia and map them to the Capability/Opportunity/Motivation-Behavior model (COM-B model).

**Results:**

Fifty-two GPs were included. The COM-B model found nine barriers to implementing dementia screening and management services in primary healthcare: (1) poor capability: lack of systematic knowledge of dementia and inadequate dementia screening skills; (2) little opportunity: unclear pathways for referral, insufficient time for dementia screening and management, lack of dementia-specific leaders, and no guarantee of services continuity; (3) low motivation: outside of GP scope, worries associated with dementia stigma rooted in culture beliefs, and insufficient financial incentives.

**Conclusions:**

Our study concluded that GPs were not yet ready to provide dementia screening and management services due to poor capability related to knowledge and skills of dementia, little opportunity associated with an unsupportive working environment, and low motivation due to unclear duty and social pressure. Accordingly, systematic implementation strategies should be taken, including standardized dementia training programs, standardized community-based dementia guidelines, expansion of primary care workforces, development of dedicated leaders, and the eradication of stigma attached to dementia to promote dementia screening and management services in primary care.

**Supplementary Information:**

The online version contains supplementary material available at 10.1186/s12877-023-03756-x.

## Background

Dementia has become a global public health challenge. As the country with the largest number of people with dementia, China accounts for 20% of the total number of dementia patients worldwide, with a current patient population of approximately 15.07 million which is expected to increase to 35.98 million in 2050 [1, 2]. Alongside the rapid aging of the Chinese population, the prevalence of dementia in China has shown a significant upward trend in the past three decades. From 1990 to 2016, the age-standardized prevalence of dementia in China increased by 5.6%, while the global prevalence increased by only 1.7% during the same period [3]. Dementia is the main cause of disability and dependence among older people [4], imposing a heavy disease burden on patients, families, and society. The total annual cost of dementia in China is expected to increase from $900 million in 1990 to $114.2 billion in 2030 [5].

Faced with the public health challenges posed by dementia, many international professional associations have advocated timely screening and early intervention for dementia based on primary care facilities, since this approach may effectively alleviate the progression of the disease, reduce the incidence of complications and mortality, and thereby mitigate the social burden of dementia [6, 7]. In China, general practitioners (GPs) can provide public education, risk assessment, non-pharmacological interventions, and coordination of social and medical resources for the prevention and management of dementia [8]. Regrettably, while Chinese GPs mostly agree to provide dementia screening and management services, only a few put them into practice [9, 10]. In China, less than 10% of patients with dementia were diagnosed through GPs' dementia screening services [11], much lower than 40% in Germany [12] and 96% in England [13]. Moreover, a considerable proportion of GPs do not recognize screening and managing dementia as part of their duties, and they are unwilling to or even do not provide dementia diagnosis and treatment services [9, 14, 15]. The findings of previous studies have proven that lack of knowledge of GPs [9, 14], insufficient cognition and concern of patients and their families [16–18], unclear referral mechanisms [10, 19, 20], insufficient time for clinical consultations, and inapplicability of screening tools [10, 21, 22] are the barriers for GPs to practice dementia screening and management services. However, there is a paucity of literature on the practical barriers encountered by Chinese GPs in the provision of dementia screening and management services with only a few studies on GPs' perceptions of developing dementia services, and their knowledge, attitudes, and skills of screening and managing dementia [9, 10, 14]. The city evaluated in this study has shown rapid progress in the construction and implementation of a social and psychological service system for older adults, and fully implemented the community dementia prevention and treatment services program based on community health service centers (CHSCs) in 2021. Thus, exploration of the behavioral logic underlying the gap between the vision of CHSCs-based dementia screening and management services and its adoption by GPs in this city can provide lessons for other urban CHSCs that are developing dementia screening and management services. Research suggests the use of theoretical frameworks can be most effective when understanding behavior, with the Capability/Opportunity/Motivation-Behavior model (COM-B model) being a recommended approach [23]. The COM-B model regards the specific behaviors of a given population as a "behavioral system", and the basic conditions for such behaviors include capability (the individual's psychological and physical ability), opportunity (all the factors that lie outside the individual's control), and motivation (the mental processes that energize and direct individual behavior) [23]. No studies have used the COM-B model to understand GPs' dementia screening and management behavior. Therefore, using the COM-B model, this study aimed to employ a qualitative approach to explore the reasons that hindered the provision of dementia screening and management services by GPs in general practice.

## Methods

### Study design

This study used a qualitative research design to explore the factors that hindered the provision of dementia screening and management services by GPs. The in-depth interviews and focus groups were used for different information from the participants. While focus groups can generate a wider range of ideas and perspectives than in-depth interviews [24], and in-depth interviews capture more detail and provide greater insight into participants' ideas and experiences than focus groups [25]. This study was reported according to the Consolidated Criteria for Reporting Qualitative Research (COREQ) checklist (Additional file 1).

### Setting and participants

This study was conducted in a first-tier city in southern China that has shown a "pioneering demonstration" of social and economic development. After reforming the primary health care system, the city established a comprehensive medical service network, and its community health service management system is coordinated and standardized by hospitals and managed by subordinate community health service management centers. In 2021, the city's Health Commission required CHSCs to provide dementia prevention and treatment services following the "National Basic Public Health Service Specification (Third Edition)". In addition, as the city with the largest proportion of migratory population, the high mobility of older adults migrating with their children introduced new challenges for timely screening and effective management of dementia in CHSCs.

A purposive sampling process was used. Participants were recruited through a list of GPs' dementia skills training sessions that included information on the names, addresses, and phone numbers of sixty GPs from fifty CHSCs. All GPs have provided dementia screening and management services. The researchers invited thirty-five GPs to participate in the focus groups by telephone or text message, five of whom declined due to lack of time. The other thirty GPs accepted the invitation and none withdrew. After the focus groups, another twenty-five GPs on the list who had not participated in the focus groups were invited for in-depth interviews and all agreed to participate.

### Data collection

Data collection took place face-to-face from September 2021 to January 2022. This research team is a multidisciplinary team consisting of a professor of anthropology(NG), a professor of public health(JL), a nursing postgraduate supervisor(WJC), a community public health practitioner(JFZ) and three nursing postgraduates(DY、QYH、LH), all of whom have experience in qualitative research, and all are female, except NG and JFZ. As a research team focused on aging, it is particularly concerned about the low identification rate of dementia in primary care, while the dementia screening and management behavior of GPs is crucial for the patients with dementia. The researchers interpreted qualitative data differently depending on their field of study and have a more holistic view of the results than researchers from a single discipline. None of the researchers knew the participants before the study. Three focus groups involving thirty GPs were conducted. Each focus group consisted of ten GPs, and lasted for 90 min. All focus groups were conducted in conference rooms with the assistance of three researchers, including a facilitator, a recorder, and an observer, to capture verbal and non-verbal information. The number of participants for focus groups was based on the standard recommendations for focus groups [26]. The interview guide of focus groups was developed based on the literature to investigate GP dementia practice, difficulties encountered and attitudes towards dementia screening and management services in CHSCs (Additional file 2) [10, 22]. All interviews were completed by DY. The guide for the interviews was developed using the COM-B model to explore the barriers to GPs' dementia screening and management services (Additional file 3) [23]. The interviews were conducted at an appropriate time after participants had completed their work in their consultation rooms. The interviews were collected until information saturation was reached, determined when no new ideas emerged from the interviews. The twenty-two interviews were completed and lasted between 20 to 75 min (average duration, 46 min). All interviews and focus groups were audio-recorded and transcribed verbatim by the interviewer within 24 h of the end of the interview and were treated anonymously.

### Data analysis

Data collection and data analysis were conducted simultaneously. All transcripts were imported into NVivo12 for data management and analysis. The data of in-depth interviews and focus groups were analyzed using deductive thematic analysis. The COM-B model was used as a framework to categorize the emerging sub-themes [23, 27]. Initially, two researchers (DY, JFZ) independently assigned the initial codes by repeatedly listening to the recordings and reading the transcripts and field notes. The codes from the previous step were sorted into the sub-themes. Codes and emerging sub-themes were then reviewed and discussed with researchers (NG, JL). Secondly, a deductive framework analysis was performed. Those sub-themes were mapped under the COM-B model components (capability, opportunity, and motivation). This stage of analysis was completed by DY under the guidance of researcher JL, who has experience in COM-B model research. Finally, all researchers discussed and reviewed the themes together until an agreement was reached. Table 1. showed an example of mapping the sub-themes to the COM-B model in this study.

**Table 1:** Examples of Mapping the sub-themes to the COM-B Model

Theoretical domain	Sub-theme	Coding	Meaning unit
Capability（Psychological Capability）	Lack of systematic knowledge of dementia	Insufficient knowledge of the symptoms of the disease	You need specialist knowledge, such as symptoms, complications and.....
		Misconceptions about the disease	Dementia's disease is forgetfulness.
		Insufficient knowledge of the treatment of the disease	What can I do for the management? And what medication can they take?
Capability（Physical Capability）	Inadequate dementia screening skills	Difficulties in using assessment scales	A lot of GPs don't know how to ask and should be trained.
		Irregular application of assessment tools	Asking questions in a conversational manner should not be essential for every item

### Ethical consideration

This study was conducted according to the guidelines of the Declaration of Helsinki and approved by the Institutional Review Board of the First Affiliated Hospital of Jinan University (Ethical approval number KY-2022–053).

## Results

### Participants characteristics

A total of 52 GPs (26 males and 26 females, mean age 31.3 years, average working experience 6.6 years) were recruited. Most of the participants had a master's degree. The participant characteristics were summarized in Table 2, and detailed data for participant characteristics could be found in Additional file 4.

**Table 2:** Participant characteristics

Characteristic	Category	No. of participants
Sex	Male	26 (50%)
	Female	26 (50%)
Age (mean, range)		31.3 (24-46)
Years in practice (mean, range)		6.6 (1-28)
Education level	Below Bachelor's degree	6 (11.5%)
	Bachelor's degree	21 (40.4%)
	Master's degree	25 (48.1%)

### Qualitative findings

**Fig. 1:**
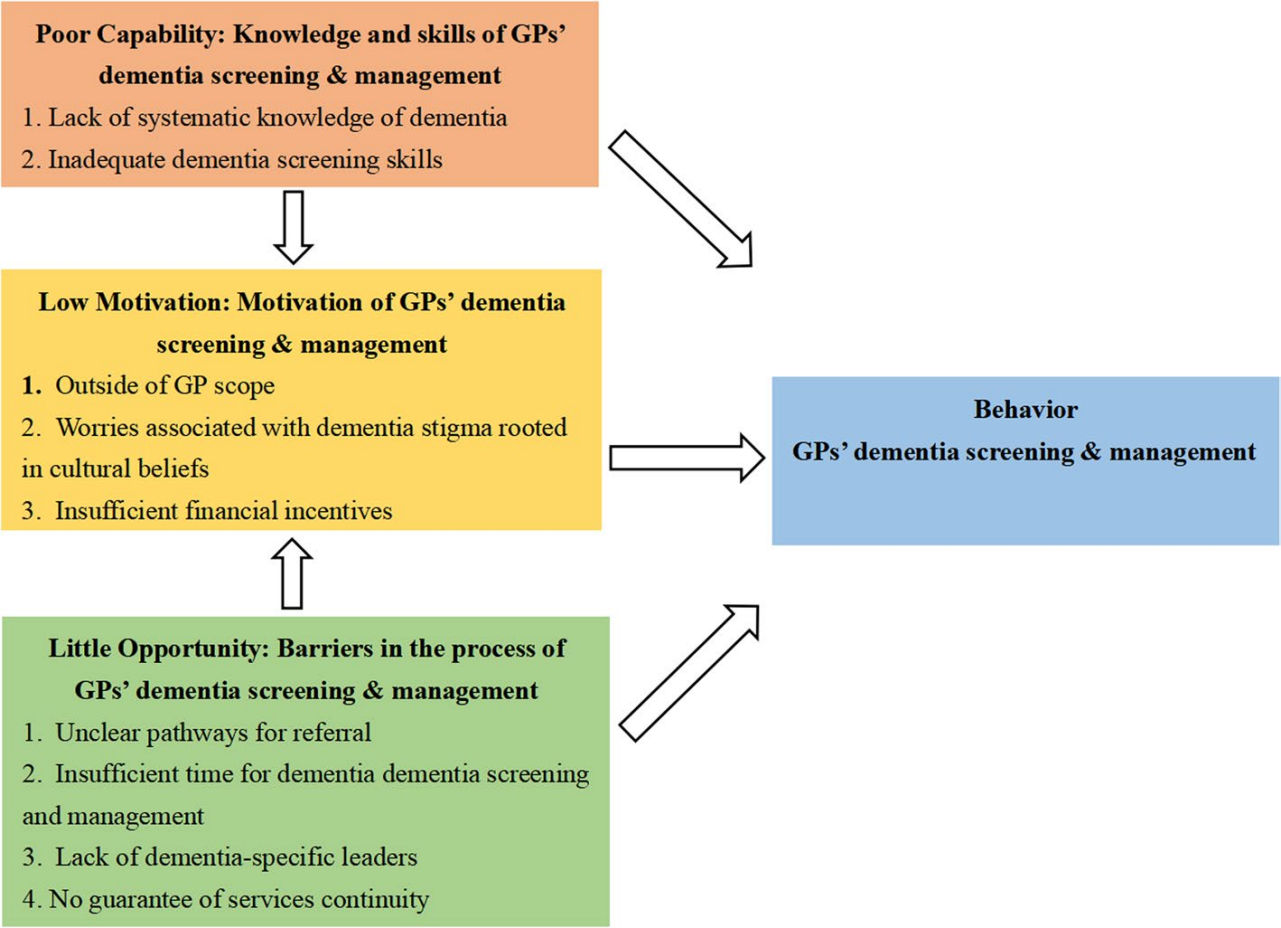
Barriers to dementia screening and management based on the COM-B model

As shown in Fig. 1, using the COM-B model, the results of this study were analyzed in terms of the dimensions of capability, opportunity, and motivation, and nine barriers affecting the behavior of GPs in providing dementia screening and management services were found. The specific findings for each of these barriers and some examples of participant responses outlining these barriers were described in the sections below.

### Poor Capability (C): Knowledge and skills of GPs' dementia screening and management

GPs generally believed that their capability for screening and managing dementia was far from sufficient, which mainly manifested as a lack of systematic dementia knowledge and inadequate skills in dementia. This lack of capability made GPs equate the screening and managing dementia to the experience of "groping for stones to cross a river".

### Lack of systematic knowledge of dementia

 More than three-quarters of GPs reported that they had limited knowledge of dementia. In addition to being unable to clearly describe the stages of dementia as well as the symptoms and complications of each stage, GPs also equated the cause of dementia with age-related brain degeneration. This "knowledge gap" made it difficult for GPs to provide dementia screening and follow-up services to older adults.

"It requires professional knowledge of aspects such as premenstrual symptoms, disease progression, complications, etc." (FG:GP 20, female).

In addition, due to their lack of clarity about high-risk factors and diagnostic criteria for dementia, GPs questioned their diagnosis and treatment results, which even affected their diagnosis and treatment records for older people.

"What are the initial symptoms of dementia in the early stage, what are the characteristics of high-risk groups, and what are the criteria for diagnosis and treatment? I don't know how to judge the stage of dementia in older people and to document the condition." (FG:GP 23, male)

### Inadequate dementia screening skills

 Gaps in dementia knowledge further contributed to GPs not having the skills for dementia screening services. The AD8 is used as a cognitive function assessment tool in Chinese CHSCs. Most GPs reported they were not skilled in using the dementia screening tools and it was particularly challenging to translate the scale items into questions that were easy for older adults to understand.

"The key points are time and place disorientation, lack of computing skills, and so on. How do you switch questions to get what you want when the patient doesn't understand, and how do you switch the questions to make sure that the content is consistent?" (I:GP 36, male).

Some GPs even selectively evaluated some items or determined the cognitive function status of older adults by relying on experience rather than dementia tests.

"All assessments are based on subjectivity. Asking questions in a conversational manner should not be essential for every item, and cognitive function is then rated on the basis of my subjective feelings." (I:GP 33, male).

This phenomenon of "nonstandard" dementia screening, including incomplete implementation of assessment scales or non-implementation of assessment scales, was prevalent in GPs' practice. Thus, negative dementia screening practice had become a "routine" for GPs.

### Little Opportunity (O): Barriers in the process of GPs' dementia screening and management

Although dementia screening and management services are available in CHSCs, the mismatch of supportive resources within the medical system does not facilitate GPs to provide dementia screening and management services for older adults. Most importantly, there is no specific community guideline for dementia prevention and treatment that provides GP with the evidence-based advice on dementia treatment, care, and support. Thus, even when GPs detected suspected patients with dementia or cognitive impairment, there was no clear guideline for them to refer these patients. In addition, almost all GPs indicated that the cost of screening and managing dementia, including the time cost and unavailability of dementia-specific resources, was the main barrier in terms of external environmental resources. And GPs also reported that the migratory aged population accounted for the majority of the older adults and continuity of screening and managing dementia could not be guaranteed for this population. As a result, GPs lacked adequate opportunities to carry out dementia screening and management services in the face of competing daily clinical tasks.

### Unclear pathways for referral

 The process of dementia diagnosis is complicated. GPs believed the diagnosis of dementia was a vague concept without guidelines for dementia diagnosis and management. They played the role of "discoverers" in the screening and managing dementia but not served as "guides" for dementia patients.

"(referral) vague concept. The pathway is unclear as to where exactly to go and what to do next for the positive patients (who screen out)." (I:GP 33, male).

Some GPs responded that older people complained that the detection process for dementia was too complicated and that they could not get a diagnosis at CHSCs and needed to go to a higher-level hospital for further screening and testing.

"Some older adults find it troublesome to go to a higher-level hospital for screening and examination after screening here, and they want to get accurate results.” (FG:GP15, male).

In addition, the management of dementia requires medical advice from specialists. The lack of referral mechanisms between CHSCs and tertiary hospitals prevented GPs from engaging in the community management of dementia.

"Where should the positive patients go for follow-up tests? There is no specific department to cooperate with us, and we only have an obligation to inform them that they can go to the neurology department of a large hospital. Moreover, there is no way to trace whether the patient went or not because there is no interconnected interaction with the higher-level hospitals. " (I:GP 36, male).

### Insufficient time for dementia screening and management

 A complete dementia screening procedure includes an assessment of cognitive function, evaluation of medical history, and health education on dementia prevention and treatment. Due to the heavy workload and tight working schedules of CHSCs, GPs believed that dementia screening and management services were time-consuming for patients with multiple health problems. Thus, dementia screening and management was often ignored by GPs as a secondary examination due to time pressures.

"Dementia screening is a time-consuming task. The CHSC is too busy to talk to older people about dementia or for health education. The main problems are hypertension and diabetes or something else. We don't have time to dedicate to this work. It is a problem of time allocation…this is the root." (FG:GP 01, male).

Some GPs suggested the provision of a special time for screening and managing dementia through the appointment system, which would allow them to adequately complete dementia-related services of the target population.

"The evaluation based on the AD8 scale assessment does not take much time, but you also need to ask about the patients' previous history. The high-risk patients or AD8-positive patients require further evaluation and health education and should make an appointment with a doctor." (I:GP 38, male).

### Lack of dementia-specific leaders

 Another cost barrier to screening and managing dementia mentioned by GPs was the lack of dementia-specific talent resources. In the management model of patients with mental disorders in CHSCs, GPs can link to social resources and other medical institutions to achieve multidisciplinary management of patients by systematic training [28]. Likewise, GPs believed that setting up dementia specialists would reduce their workload and optimize the management of people with dementia in the overcrowded CHSCs.

"We can do it together because of the large workload of dementia screening. If the people with dementia who are screened out are assigned by a dedicated person for management, the service will be refined." (FG:GP 05, female).

### No guarantee of services continuity

 The older adults served by the CHSCs are mainly migratory older individuals called the "floating" aged population. GPs mentioned that there was no guarantee of regular dementia screening for such migratory-aged populations, and the predetermined benefits of large-scale dementia screening would be difficult to achieve in this population.

Older people do not develop dementia at once, and they have to be followed up continuously. They are very mobile, just like 'migratory birds'. They don't come here every year, so they can't be screened several times." (I:GP 34, female).

### Low Motivation (M): Motivation for GPs' dementia screening and management

The limitations in GPs' capability and opportunity to screening and managing dementia also affected their willingness to provide dementia screening and management services, and their unwillingness led them to perceive dementia screening and management skills as having low efficacy. Moreover, some GPs were less motivated to participate in dementia-related services because of the psychological pressure caused by the negative emotions arising from talking to older adults about dementia during their consultations. And financial incentives were cited as the biggest motivator to perform dementia-related services.

### Outside of GP scope

 GPs considered the detection and follow-up of dementia to be relatively complex and within the domain of the department of neurology. Due to the lack of capability in screening and managing dementia and authority to prescribe dementia medication, GPs could only provide dementia screening services and non-pharmacological interventions. In addition, they perceived a lower sense of efficacy in screening and managing dementia in comparison with chronic diseases such as hypertension and diabetes. Therefore, GPs were not in favor of assigning complex disease tasks to low-skilled primary care practitioners.

"We can't diagnose without qualification. How can we talk to them about dementia? Of course, they should see a specialist to confirm the diagnosis. Dementia must be professionally managed, just like the management of mental disorders, where you need to go through professional training to be able to call yourself a 'community psychiatrist' to manage your patients." (FG:GP 02, female).

At the same time, the lack of confidence in their professional ability also made GPs afraid of misdiagnosis.

"We can't interpret EEGs and the results of body fluid tests. Our professional ability is not as high as that of specialists, so we are afraid of missed diagnosis." (FG:GP 16, male).

### Worries associated with dementia stigma rooted in culture beliefs

Discussing dementia with older individuals openly is considered a taboo in the Chinese socio-cultural context. The GPs reported that older adults equated dementia with negative labels such as "neuropathy", "abnormal thinking", and "sickness". Older people showed commonly negative emotions such as resistance and fear when GPs offered dementia screening to them. These negative emotions might inhibit older people from participating in dementia screening and cause them to conceal their cognitive function status, reducing the credibility of the assessment and thereby causing psychological pressure on the GPs.  

"Older people avoid talking about dementia. They get scared when they hear about dementia or you give them a dementia screening. Even if I feel that older people have cognitive problems, they will deny it, reject it, and be reluctant to admit it. The patient's and the family's awareness are not strong enough, and many things (disease symptoms) are covertly ignored as a result. How do I continue?" (FG:GP 05, female)

When GPs performed dementia screening, they used the term "health screening" or "cognitive screening" rather than "dementia screening" to avoid causing negative emotions in older adults.

"Usually, we say cognitive survey or do a health survey. If you talk about dementia screening, the older patient will be unhappy to hear it, and may think is there something wrong with him/her and will be repelled." (I:GP 33, male).

### Insufficient financial incentives

 The financial incentive was a key motivator repeatedly mentioned by GPs in relation to screening and managing dementia, and was one of the reasons for driving further visits among screen-positive older people. However, with little or even no remuneration for dementia screening and management in CHSCs, GPs were not motivated to proactively provide dementia screening and management services until people with dementia had developed behavioral and psychological symptoms of dementia.

"Wherever you want people, you have to invest money. If we are not remunerated for undertaking dementia-related services, we won't have motivation. Policies and benefits need to keep up." (FG:GP 05, female).

"There are also older adults with dementia who have developed psychiatric symptoms, and they are included in the management of mental disorders. The management of six major categories of mental illness is included in the scope of national public health management. Psychiatric symptoms caused by dementia are included in psychiatric management. There is no good treatment for the early stage of dementia and mental decline." (FG:GP 01, male).

Therefore, the respondents recommended incentivizing GPs to allocate more time and effort to dementia screening and management services through performance-based subsidies.

"We are remunerated for managing chronic diseases and mental illnesses. How is the workload calculated if I do this work without seeing the patients? Without performance, no one is motivated to do it." (FG:GP 01, male).

## Discussion

This study aimed to explore barriers in the provision of dementia screening and management services by GPs. Our results suggested that despite being called upon for dementia prevention and treatment, GPs could not fulfill their key role in dementia prevention and treatment due to limitations in terms of capability, opportunity, and motivation. This situation had led to a passive involvement of GPs in screening and managing dementia and less priority of dementia-related services.

The results of this study showed that GPs often did not have sufficient theoretical knowledge and practical skills for providing effective dementia-related services. Their inadequate capability limited GPs' dementia practice, causing delayed diagnosis, undertreatment, and misunderstandings about behavioral and psychological symptoms of dementia (BPSD). Dementia screening and management services include the provision of counseling, dementia screening, and health education and promotion [29]. The complexity of the biomedical and psychosocial aspects of screening and managing dementia requires GPs to have an understanding of dementia pathology, methods of treatment and care, and diagnostic processes. However, there is a clear knowledge gap among health staff in the Chinese community [30, 31], which stems from a serious lack of dementia training. First, dementia education is not included in medical professional education and training programs, and dementia skill training is concentrated in the departments of neurology, psychiatry, and geriatrics in general or specialized hospitals [30, 32, 33]. Second, continuing dementia education for CHSC workers is often transient or short-term, and has little impact on treatment practice [10, 34].

In addition to the barriers to capability, barriers to opportunity, which were beyond the individuals' control, further affected GPs' dementia screening and management practice. These barriers to opportunity included insufficient time, inadequate labor resources, mobility of the screened individuals, and lack of collaboration among medical institutions. In the context of the Chinese primary care system, GPs are overloaded because they have to simultaneously perform medical tasks and provide public health services [35, 36], leaving them with no energy to focus on dementia-related services. For example, standardized dementia screening procedures cannot be completed in a single consultation, and clarification of the differential diagnosis of dementia is challenging due to time constraints [22, 37]. With the normalization of efforts for prevention and control of the COVID-19 epidemic, GPs require additional time and effort to provide dementia screening and management and are not supported by the current disease and task performance-oriented healthcare culture [35]. Only well-trained GPs can follow the dementia guidelines more closely and optimize the dementia diagnosis and treatment plan to ensure the quality of services [38]. In addition, multidisciplinary collaborations and interactions in primary care settings can facilitate timely referrals and early interventions for people with dementia [39, 40]. However, like other developing countries, community dementia screening and management in China are in the exploratory stage, with an underdeveloped system for community-based dementia diagnosis and support and no coordination between primary and specialist care facilities [10]. Notably, this study was conducted in the city with the largest proportion of migrant seniors in China. The migratory of these older patients precluded them from accessing continuous services and reduced their compliance when they received dementia care services [41], all of which made the documentation, follow-up, and management of health services, including dementia-related services, more difficult for these older patients than for the local aged population.

The abovementioned barriers to capability and opportunity further inhibited GPs' motivation to provide dementia screening and management services. First, the lack of capability led to low subjective self-efficacy among GPs and caused them to question the value of the timely diagnosis of dementia. Such negative beliefs and attitudes could profoundly affect GPs' dementia diagnosis and management practices [42]. As a result, they often lacked confidence in the diagnosis and management of dementia [42, 43], particularly for patients with BPSD [44], and did not agree with their responsibilities in screening and managing dementia [38]. As shown in the results of this study, the respondents believed that their qualifications in dementia were not as good as those of experts and therefore questioned the role of GPs in dementia prevention and treatment. This was different from the findings in Changsha and Beijing, China [9, 14], where GPs were generally enthusiastic about developing dementia screening and management services in the future. This difference may be attributed to the fact that our survey was conducted during the COVID-19 epidemic, and the workload and focus of GPs' work were different before and during the epidemic. Second, the availability of specialists and diagnostic services can improve GPs' willingness to engage in dementia screening and management [42], but these resources  are not readily available in reality. Furthermore, the importance of incentives has been well recognized. Remuneration can provide GPs with the necessary incentive to adopt dementia screening and management services [38], encourage GPs to actively participate in community dementia management, and ensure continuity of services [45]. However, primary health care in China is focused on chronic and infectious diseases such as diabetes, hypertension, and tuberculosis, and dementia is not included in the list of performance indicators. Since GPs' work is naturally performance-oriented, they rarely show initiative for managing specific diseases that they are not required to by the government [11]. In addition, unlike other diseases, the diagnostic logic of dementia in GPs is influenced by non-medical factors such as moral/ethical considerations, wishes of the patient/family, and social inclusion for people with dementia [42], and their treatment decisions are more complex and individualized. Moreover, stigmatizing attitudes toward dementia continue to hinder timely diagnosis and services utilization in the community [11, 46], causing older people to deliberately hide information about their cognitive status [38] and potentially affecting the accuracy of assessment results. As a result, GPs face more psychological pressure when treating people with dementia. To address this situation, GPs avoid using clinical terms such as "dementia screening" and "dementia" in their consultations, and such inappropriate interview techniques will exacerbate the gap in the public's correct perception of dementia and are not conducive to public education on dementia.

Therefore, based on the above barriers of capability, opportunity, and motivation that affect behavior, GPs' enthusiasm for providing dementia screening and management services gradually faded and they coped with dementia work by avoiding work inertia. Especially in the overloaded work status during the COVID-19 epidemic, GPs could not balance various emergent and routine tasks and tended to prioritize more urgent tasks as well as tasks in which they are more competent. In this context, dementia screening and management services were regarded as "thankless tasks" and gradually "non-existent".

### Strengths and limitations

The strength of this study lied in the use of the COM-B model to guide data collection and analysis. The conceptual analysis of barriers to GP dementia screening and management through this model was critical for understanding the realities of GP-based dementia screening and management services and for developing solutions for changing GP dementia practice. In addition, the participants were from a city in southern China. As a pilot demonstration area for the "Healthy China" program, the modernization of this city's medical and health governance system has become a proof of practice for other regions, and it has launched the first community-based dementia prevention and treatment programs. Thus, our results provided unique evidence for other efforts to plan and implement dementia screening and management in communities. Finally, in-depth interviews and group discussions were used for data collection, which yielded both broad and focused opinions from the participants.

This study also had several limitations. First, the predetermined analytical framework might have led us to miss important factors outside of the COM-B model. In addition, due to the ambiguity of the data, text units might involve more than one theoretical domain. We coded the meaning units into the domains that we deemed to best reflect the key themes, as suggested by the developers of COM-B [47]. Second, the participants were all from one city, and their opinions do not represent the views of all GPs. And, this study did not focus on other health workers in CHSCs. Moreover, considering differences in the forms, content, and approaches for dementia screening and management in CHSCs in different cities and regions, the findings of this study could only be applied to some primary care settings. Finally, the addition of dementia screening and management services to the CHSCs in this study was late, and most GPs had insufficient experience in screening and managing dementia, which might have contributed to the bias in the results.

## Conclusion

This study used the COM-B model to provide a preliminary exploration of the practice gap for dementia screening and management services provided by Chinese GPs. Our findings showed that barriers related to capability, opportunity, and motivation prevented GPs from playing their role in the frontline of dementia care even if they were asked to carry out dementia screening and management services. These diverse and multi-layered barriers indicated the need for complex solutions, including standardized dementia training programs, standardized community-based dementia guidelines, expansion of the primary care workforces, development of dedicated leaders, and universal dementia health education to bridge the gap between the reality and the expectations of dementia screening and management services of CHSCs.

### Implications for practice and policy

The findings of this study have some research and policy implications for China. First, the capability of GPs in screening and managing dementia should be strengthened. Include dementia management in GP training courses and train GPs in dementia diagnosis and disease management skills, including knowledge of pathophysiology, use of screening tools, diagnosis, and treatment methods. Secondly, a comprehensive community dementia treatment system should be established. Relevant government departments could design special programs for dementia prevention and treatment based on supporting resources in the community. Service resources such as specialist medical institutions, community health service centers, rehabilitation agencies, and social worker agencies should be integrated to create good conditions for community health service workers. Finally, the inclusion of dementia in primary public health services and increased financial investment will facilitate dementia prevention and treatment in CHSCs. At the same time, expanding human resources, especially mental health professionals, is particularly important for the overall management of dementia.

## Supplementary Information

**Additional file 1.** COREQ checklist

**Additional file 2.** Interview Guide of focus group

**Additional file 3.** Interview Guide of in-depth interviews

**Additional file 4.** Characteristic of participants

## Data Availability

The datasets generated and/or analyzed during the current study are not publicly available in the interest of participant privacy and confidentiality but are available from the corresponding author on reasonable request.

## Abbreviations

GP: General practitioner
CHSC: Community health service center
COM-B model: Capability/Opportunity/Motivation-Behavior model
COVID-19: Coronavirus disease 2019
BPSD: Behavioral and psychological symptoms of dementia
FG: Focus group
I: Interview
