# Supplementary material for: Exploring barriers to dementia screening and management services by general practitioners in China: a qualitative study using the COM-B model
Source: BMC Geriatr. 2023 Jan 31;23:55. doi: 10.1186/s12877-023-03756-x (PMC9886538; doi:10.1186/s12877-023-03756-x)
Supplement: Supplementary file 1 — Additional file 1. COREQ checklist [file 12877_2023_3756_MOESM1_ESM.pdf]

Consolidated Criteria for Reporting Qualitative Research (COREQ): a 32-item checklist

Please indicate in which section each item has been reported in your manuscript. If you do not feel an item applies to your manuscript, please enter N/A.

For further information about the COREQ guidelines, please see Tong *et al.*, 2017: https://doi.org/10.1093/intqhc/mzm042

No.	Item	Description	Section #
**Domain 1: Research team and reflexivity**			
Personal characteristics			
1.	Interviewer/facilitator	Which author/s conducted the interview or focus group?	NG, JL, DY, JFZ, LH, and QYH
2.	Credentials	What were the researcher's credentials? *E.g. PhD, MD*	NG- PhD<br>DY- BD<br>JFZ- BD<br>QYH- MD<br>LH- BD<br>WJC- BD<br>JL- PhD
3.	Occupation	What was their occupation at the time of the study?	NG- Associate professor<br>DY- Postgraduate student<br>JFZ- Postgraduate student<br>QYH-MA<br>LH- Postgraduate student<br>WJC-Professor<br>JL- Associate professor
4.	Gender	Was the researcher male or female?	All are female, except NG and JFZ.
5.	Experience and training	What experience or training did the researcher have?	NG- Completed a large number of qualitative studies<br>DY- Community nursing internship experience<br>JFZ- Experience in medical statistics research<br>QYH- Experience diabetes nursing research<br>LH- Community nursing internship experience<br>WJC- Rich experience in nursing management and diabetes nursing research<br>JL- Led a number of healthy aging projects, including community-based health management<br>All of whom have experience in qualitative research and JL has experience in COM-B model research.
Relationship with participants			

6.	Relationship established	Was a relationship established prior to study commencement?	We didn't establish a relationship with participants prior to study commencement.
7.	Participant knowledge of the interviewer	What did the participants know about the researcher? *E.g. Personal goals, reasons for doing the research*	Informed Consent Statement
8.	Interviewer characteristics	What characteristics were reported about the interviewer/facilitator? *E.g. Bias, assumptions, reasons and interests in the research topic*	This research team are particularly concerned about the low identification rate of dementia in primary care, while the dementia screening behavior of GPs is crucial for the timely identification of patients with dementia.
**Domain 2: Study design**			
Theoretical framework			
9.	Methodological orientation and theory	What methodological orientation was stated to underpin the study? *E.g. grounded theory, discourse analysis, ethnography, phenomenology, content analysis*	thematic analysis
Participant selection			
10.	Sampling	How were participants selected? *E.g. purposive, convenience, consecutive, snowball*	purposive sampling
11.	Method of approach	How were participants approached? *E.g. face-to-face, telephone, mail, email*	telephone, text message and face-to-face
12.	Sample size	How many participants were in the study?	52
13.	Non-participation	How many people refused to participate or dropped out? What were the reasons for this?	5 refused to participate due to lack of time<br>None dropped out
Setting			
14.	Setting of data collection	Where was the data collected? *E.g. home, clinic, workplace*	conference rooms and CHSCs consultation rooms
15.	Presence of nonparticipants	Was anyone else present besides the participants and researchers?	No
16.	Description of sample	What are the important characteristics of the sample? *E.g. demographic data, date*	Table 2.
Data collection			
17.	Interview guide	Were questions, prompts, guides provided by the authors? Was it pilot tested?	Additional file 2, 3.<br>The interview guide was not pilot tested.

18.	Repeat interviews	Were repeat interviews carried out? If yes, how many?	No
19.	Audio/visual recording	Did the research use audio or visual recording to collect the data?	The research used audio recording to collect the data.
20.	Field notes	Were field notes made during and/or after the interview or focus group?	focus group: Yes.<br>Interview: No.
21.	Duration	What was the duration of the interviews or focus group?	Yes
22.	Data saturation	Was data saturation discussed?	Yes
23.	Transcripts returned	Were transcripts returned to participants for comment and/or correction?	No
Domain 3: analysis and findings			
Data analysis			
24.	Number of data coders	How many data coders coded the data?	Four
25.	Description of the coding tree	Did authors provide a description of the coding tree?	Yes, in Table 1.
26.	Derivation of themes	Were themes identified in advance or derived from the data?	Derived from the data
27.	Software	What software, if applicable, was used to manage the data?	NVivo 12
28.	Participant checking	Did participants provide feedback on the findings?	No
Reporting			
29.	Quotations presented	Were participant quotations presented to illustrate the themes / findings? Was each quotation identified? *E.g. Participant number*	Yes, themes are presented using quotes.<br>All quotations were identifted by numbers and gender. No more details were added due to confidentiality.
30.	Data and findings consistent	Was there consistency between the data presented and the findings?	Yes. In the result section,quotations are used to illustrate the consistancy.
31.	Clarity of major themes	Were major themes clearly presented in the findings?	Yes
32.	Clarity of minor themes	Is there a description of diverse cases or discussion of minor themes?	Yes, within the selected data diverse cases are described and minor themes discussed.

Developed from: Allison Tong, Peter Sainsbury, Jonathan Craig, Consolidated criteria for reporting qualitative research

(COREQ): a 32-item checklist for interviews and focus groups, International Journal for Quality in Health Care, Volume 19, Issue 6, December 2007, Pages 349–357, https://doi.org/10.1093/intqhc/mzm042
