# Supplementary material for: Exploring barriers to dementia screening and management services by general practitioners in China: a qualitative study using the COM-B model
Source: BMC Geriatr. 2023 Jan 31;23:55. doi: 10.1186/s12877-023-03756-x (PMC9886538; doi:10.1186/s12877-023-03756-x)
Supplement: Supplementary file 2 — Additional file 2. Interview Guide of focus group [file 12877_2023_3756_MOESM2_ESM.pdf]

Interview guide of focus groups

Core Question	
1. Does your CHSC provide dementia screening and management services?	Yes<br>①How do you approach dementia screening and management services?<br>② How do you organize this task?<br>③What is the specific process for providing dementia screening and management services?<br>④ What is your specific assignment?<br>⑤ How did you proceed with this task?<br>⑥Are there any experiences worth recommending?
	No<br>① When are you planning to launch?<br>②What did you do to prepare before the launch?
2. What is the significance/purpose of dementia screening and management in your opinion?	① Can you achieve your goal with dementia screening and management? Why?<br>②Do you approve of dementia screening and management in CHSCs? Why?
3. How do you think dementia screening and management is similar to other screenings?	① What's the difference?
4. What do you think of the effectiveness of AD8 as a primary screening tool?	① Does the AD8 scale work? Why?
5. How do you plan to conduct further management and arrangements for older people who screen positive?	
6. What are the difficulties in dementia screening and management services?	① What solutions can you think of?<br>② What resources and support are available to help you run a successful dementia screening and management?
7. What are some possible solutions?	

Supplementary question.

8. Do you think CHSC dementia screening and management offers any advantages over screening and management by hospitals?

9. How does the existing consultation system support GPs to screen older people for dementia?

10. Do you have any suggestions regarding the current specific process of dementia screening and management by the Social Welfare Department?
