# Supplementary material for: Exploring barriers to dementia screening and management services by general practitioners in China: a qualitative study using the COM-B model
Source: BMC Geriatr. 2023 Jan 31;23:55. doi: 10.1186/s12877-023-03756-x (PMC9886538; doi:10.1186/s12877-023-03756-x)
Supplement: Supplementary file 3 — Additional file 3. Interview Guide of in-depth interviews [file 12877_2023_3756_MOESM3_ESM.pdf]

Interview guide for in-depth interviews

Capability

1. What knowledge do you use in providing dementia screening and management services for the elderly?
2. What other areas of knowledge do you think you lack?
3. What skills do you think GPs need to provide dementia screening and management?
4. What skills do you have?
5. What skills do you need to improve? Why? How?
6. How do you screen older people for dementia and manage them subsequently?

Opportunity

7. What difficulties have you encountered in providing dementia screening and management services for older people?
8. What factors can prevent GPs from providing dementia screening and management services?
9. How does the health care system support you in providing dementia screening and management services?
10. What areas of support do you think need to be improved?

Motivation

11. How do you feel about the process of providing dementia screening and management services?
12. How important do you think the provision of dementia screening and management services is in the midst of your busy day-to-day work? Why?
13. How much do you agree with the role of GPs in the screening and management of dementia in older people and related services?
14. How sure are you of your knowledge and skills in dementia/how confident are you?
15. Why are you not/are you confident?
16. What support would increase your confidence in dementia screening and

management services?

17. What other suggestions do you have for GP dementia screening and management services?
