# Supplementary material for: Exploring barriers to dementia screening and management services by general practitioners in China: a qualitative study using the COM-B model
Source: BMC Geriatr. 2023 Jan 31;23:55. doi: 10.1186/s12877-023-03756-x (PMC9886538; doi:10.1186/s12877-023-03756-x)
Supplement: Supplementary file 4 — Additional file 4. Characteristic of participants [file 12877_2023_3756_MOESM4_ESM.pdf]

Characteristic of participants				
**GP No**	**Gender**	**Age**	**Education level**	**Years in practice**
FG:GP01	M	45	Bachelor's degree	20
FG:GP02	F	25	Bachelor's degree	2
FG:GP03	F	30	Master's degree	4
FG:GP04	F	32	Master's degree	6
FG:GP05	F	28	Bachelor's degree	4
FG:GP06	F	30	Master's degree	4
FG:GP07	F	45	Bachelor's degree	20
FG:GP08	M	40	Bachelor's degree	15
FG:GP09	M	30	Bachelor's degree	6
FG:GP10	M	46	Bachelor's degree	28
FG:GP11	F	28	Master's degree	2
FG:GP12	F	32	Master's degree	5
FG:GP13	M	30	Master's degree	2
FG:GP14	F	30	Master's degree	3
FG:GP15	M	28	Master's degree	2
FG:GP16	M	32	Master's degree	5
FG:GP17	M	30	Bachelor's degree	3

FG:GP18	F	29	Below Bachelor's degree	8
FG:GP19	M	42	Master's degree	12
FG:GP20	F	32	Master's degree	3
FG:GP21	M	35	Master's degree	7
FG:GP22	F	28	Bachelor's degree	5
FG:GP23	M	32	Master's degree	4
FG:GP24	F	26	Below Bachelor's degree	6
FG:GP25	F	28	Master's degree	1
FG:GP26	M	30	Master's degree	3
FG:GP27	M	35	Bachelor's degree	11
FG:GP28	M	30	Bachelor's degree	8
FG:GP29	F	29	Bachelor's degree	7
FG:GP30	M	26	Master's degree	1
I:GP31	M	28	Master's degree	2
I:GP32	F	24	Below Bachelor's degree	4
I:GP33	M	32	Master's degree	4
I:GP34	M	28	Bachelor's degree	5
I:GP35	F	30	Master's degree	3
I:GP36	M	26	Bachelor's degree	4

I:GP37	M	26	Bachelor's degree	4
I:GP38	F	30	Bachelor's degree	8
I:GP39	M	26	Bachelor's degree	4
I:GP40	M	28	Master's degree	2
I:GP41	M	32	Master's degree	5
I:GP42	F	29	Below Bachelor's degree	9
I:GP43	M	30	Master's degree	3
I:GP44	F	26	Below Bachelor's degree	6
I:GP45	F	35	Bachelor's degree	12
I:GP46	M	36	Master's degree	9
I:GP47	F	32	Bachelor's degree	10
I:GP48	F	28	Master's degree	2
I:GP49	M	33	Bachelor's degree	8
I:GP50	F	36	Master's degree	7
I:GP51	F	34	Below Bachelor's degree	13
I:GP52	F	36	Bachelor's degree	12

FG: focus group; I: interview; GP: general practitioner
